# DAXX in cancer: phenomena, processes, mechanisms and regulation

**DOI:** 10.1093/nar/gkz634

**Published:** 2019-07-27

**Authors:** Iqbal Mahmud, Daiqing Liao

**Affiliations:** Department of Anatomy and Cell Biology, UF Health Cancer Center, University of Florida College of Medicine, 1333 Center Drive, Gainesville, FL 32610-0235, USA

## Abstract

DAXX displays complex biological functions. Remarkably, DAXX overexpression is a common feature in diverse cancers, which correlates with tumorigenesis, disease progression and treatment resistance. Structurally, DAXX is modular with an N-terminal helical bundle, a docking site for many DAXX interactors (e.g. p53 and ATRX). DAXX’s central region folds with the H3.3/H4 dimer, providing a H3.3-specific chaperoning function. DAXX has two functionally critical SUMO-interacting motifs. These modules are connected by disordered regions. DAXX’s structural features provide a framework for deciphering how DAXX mechanistically imparts its functions and how its activity is regulated. DAXX modulates transcription through binding to transcription factors, epigenetic modifiers, and chromatin remodelers. DAXX’s localization in the PML nuclear bodies also plays roles in transcriptional regulation. DAXX-regulated genes are likely important effectors of its biological functions. Deposition of H3.3 and its interactions with epigenetic modifiers are likely key events for DAXX to regulate transcription, DNA repair, and viral infection. Interactions between DAXX and its partners directly impact apoptosis and cell signaling. DAXX’s activity is regulated by posttranslational modifications and ubiquitin-dependent degradation. Notably, the tumor suppressor SPOP promotes DAXX degradation in phase-separated droplets. We summarize here our current understanding of DAXX’s complex functions with a focus on how it promotes oncogenesis.

## INTRODUCTION

Oncogenic drivers promote tumorigenesis, cancer progression and resistance to therapy. This has been exemplified by well-known oncogenes such as MYC, which activates genes that control metabolism, cell proliferation and drug resistance (1–3). Efforts to discover novel oncogenic drivers can lead to new cancer therapy to improve patient outcome. It emerges that DAXX (death domain-associated protein) has potent oncogenic properties and a potential novel therapeutic target. DAXX was discovered as a FAS binding protein and a modulator of Jun N-terminal kinase (JNK)-mediated cell death in 1997 (4,5). The DAXX orthologs are only found in the animal kingdom (6). It is ubiquitously expressed in various human tissues and essential for embryonic development (7,8). It interacts with diverse proteins with functions in the cytoplasm and the nucleus. Thus, DAXX has been shown to mediate apoptosis through extrinsic death receptor pathway (4,5) as well as to regulate gene expression as a transcriptional co-repressor or co-activator by interacting with diverse DNA-binding transcription factors (TFs), epigenetic regulators, core histones and chromatin-associated proteins (9–13). The findings that DAXX contains conserved SUMO-interacting motifs (SIMs) (6,14) provide a molecular explanation for the ‘promiscuous’ interactions of DAXX with diverse proteins. As reviewed previously (15) and supported by NMR spectroscopy-based experiments (16), the SUMO-SIM interaction is an important determinant for DAXX to bind a SUMOylated protein, which can be further stabilized by additional molecular interactions. Of note, a recent proteomic survey identified nearly 7000 SUMO-modified proteins in human cells (17). Whereas conceptually DAXX could confer pleiotropic effects through interacting with diverse SUMO-modified proteins, multivalent high affinity interactions are likely important for DAXX’s specific biological functions and regulation, as exemplified by the observations that multiple weak interactions are critical for the DAXX/SPOP colocalization in the nucleus (18).

An important development was the discovery that DAXX is a chaperone for histone variant H3.3 (encoded by H3F3A and H3F3B) (19,20). This firmly places DAXX as an important chromatin regulator. Biologically, more recent studies have provided compelling evidence that DAXX can function as a tumor suppressor or an oncogene. Tumor-derived DAXX mutations have been detected in pancreatic neuroendocrine tumors (PanNETs) (21). DAXX mutations more frequently occur in the regions that interact with ATRX and the H3.3/H4 dimer (21,22), which impacts chromosome stability and telomere maintenance (23,24). Nonetheless, DAXX is rarely mutated in commonly diagnosed cancer types. As discussed below in details, increased DAXX expression has been consistently observed in diverse epidemiologically prevalent cancer types. DAXX can promote malignant phenotypes *in vitro* and tumor growth and progression *in vivo*. During the past 20 years since the cloning of the DAXX gene, there is a steady increase in our understanding of the structure of DAXX and its context-dependent functions ranging from the regulation of cell survival/death, gene expression, DNA damage repair, viral infection, to tumorigenesis. In this review, we start by summarizing the structural features of DAXX, which provides a foundation for elucidating mechanisms underlying DAXX’s functions. We then review DAXX’s oncogenic properties, followed by discussing DAXX-regulated processes and the underpinning molecular mechanisms that may contribute to oncogenesis. We conclude by an overview about how DAXX’s cellular activity is regulated.

## MODULAR STRUCTURES OF DAXX

### The DAXX helical bundle (4HB)

**Figure 1.**
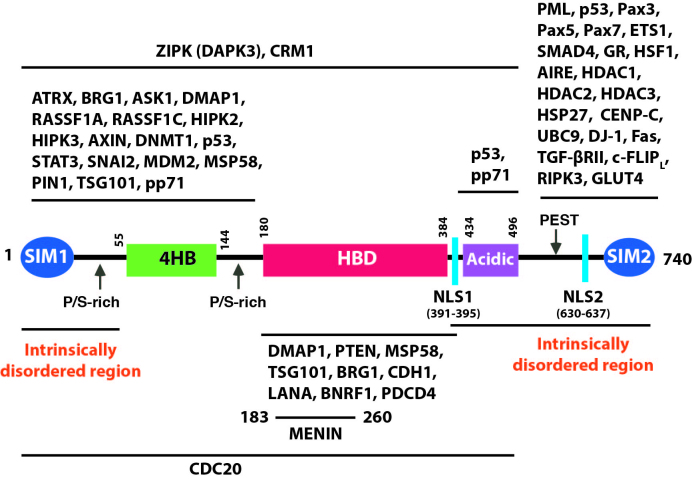
DAXX structure and interacting proteins. The modular structure arrangement of DAXX is depicted. SIM: SUMO-interacting motif, 4HB: DAXX helix bundle, HBD: histone-binding domain, NLS: nuclear localization signal. Proteins that are known to interact with specific regions of DAXX are shown. The 4HB is probably the major binding site for proteins that interact with the DAXX N-terminal region, while SIM2 likely plays a major role in binding proteins that interact with the DAXX C-terminal region. The lines below or above each protein group are not meant to be precise. Readers are referred to the original publications reporting a specific interaction (see Table 1).

DAXX consists of a modular structural arrangement with two folded structures connected by intrinsically unfolded regions (25,26) (Figure 1). A bundle of four helices spanning amino acids (aa) 55–144 is highly conserved across different species (6), which is known as DAXX helical bundle (DHB) or 4HB (22,25). It contains a defined binding surface for a number of DAXX-interacting proteins such as RASSF1C, p53 and MDM2 (25). The 4HB also contains the binding surface for ATRX, which partially overlaps the interface between DAXX and RASSF1C. The DAXX-binding domain of ATRX forms a long α-helix with a number of hydrophobic residues directly contacting DAXX 4HB (22,27,28). The DAXX–ATRX interaction is stronger than the interactions between DAXX and RASSF1C, p53 or MDM2, apparently due to additional electrostatic interactions between positively charged residues in 4HB and negatively charged residues in ATRX (22,25,27,28). Because of the overlapping binding interface between DAXX and its binding partners (ATRX, RASSF1C, p53 and MDM2), their interactions are likely mutually exclusive. As such, a ‘partner switch’ mechanism for DAXX interaction may be involved under different biological contexts (27). Intracellular membraneless bodies such as promyelocytic leukemia nuclear bodies (PML-NBs) are enriched with specific proteins and exist in a liquid-like phase that is separated from their surroundings. In addition to residing in PML-NBs, DAXX forms a phase-separated nuclear body with SPOP (18). Phase-separation could in principle favor DAXX’s interaction with a specific partner (18).

### The DAXX histone-binding domain (HBD)

The central part of DAXX spanning aa 180–397 is another highly conserved region and was termed DAXX core domain (6). This region was predicted to consist of mainly helices that could fold into a defined structure (25). The crystal structure of the core domain, also termed the histone binding domain (HBD), in complex with a H3.3/H4 dimer has been determined (26). The six α-helices within HBD wraps around the H3.3/H4 dimer, covering 40% of the surface-accessible area of the histone dimer. Structural modeling and biochemical assays show that residue G90 specific to H3.3 is the key determinant for the binding specificity of DAXX to the H3.3/H4 dimer rather than to the H3.1/H4 or H3.2/H4 dimer (26,29). Structural modeling also indicates that the DAXX HBD could only form a stable structure with the H3.3/H4 dimer but not with the H3.3/H4 tetramer, and the DAXX HBD cannot bind to the H3.3/H4 dimer in a nucleosomal context, as the N-terminal helices of the DAXX HBD in the H3.3/H4-DAXX ternary structure would severely clash with the DNA wrapped around the histone core (26). DAXX and PML enrich the H3.3/H4 dimer and ATRX in PML-NBs for histone deposition, whereas the chromatin loading of H3.3 by the histone chaperone HIRA is not affected in the absence of PML (30,31). These observations suggest that DAXX may bring H3.3/H4 to chromatin sites in conjunction with PML-NBs, whereas other parts of DAXX might mediate the dissociation of the HBD from and the release of the H3.3/H4 dimer to facilitate chromatin assembly (19,20,26). Chromatin remodelers such as ATRX and BRG1, both of which bind DAXX (32,33), may be required for the assembly of the H3.3/H4 dimer into nucleosomes in the cellular environment (19,20). Although H3.3 deposition is implicated in active transcription (34,35), DAXX/ATRX has been shown to deposit H3.3 into heterochromatin regions enriched with H3K9me3 and simple GT-rich nucleotide repeats (36).

Although the six α-helices in the DAXX HBD assemble into a stable structure when complexed with the H3.3/H4 dimer, the HBD alone is largely disordered in solution. It was shown that the formation of the H3.3/H4-DAXX HBD ternary complex promotes folding and global stabilization of all three subunits in the complex (37). Notably, a number of proteins (e.g. BRG1 and MENIN, see Figure 1) interact with the HBD. It will be interesting to study whether complex formation of the DAXX HBD with these proteins would also affect the folding of the DAXX HBD, and stabilize the corresponding complexes. Mechanistically, interactions of the HBD with other proteins could facilitate the release of histones for nucleosome assembly.

### The SIMs

Two SIMs in DAXX, located at the N- and C-terminus respectively (6,14) (Figure 1), are functionally important. Both SIMs are biochemically similar with an identical hydrophobic core flanked by acidic resides or residues that can be phosphorylated. SIM1 binds SUMOs predominantly in a parallel orientation and exhibits ∼4-fold higher affinity to SUMO1 and SUMO2 compared to SIM2 (16). SIM2 in the context of the DAXX C-terminal 20 amino acids (aa 721–740) also binds SUMO1 in parallel orientation (38). Of note, a longer SIM2 peptide of the mouse Daxx (aa 718–739) appears to bind SUMOs in both parallel and antiparallel orientations (16). Additionally, based on molecular dynamics simulations the antiparallel binding of a SIM with SUMO results in a complex with higher stability compared to the parallel orientation (39). The Nup358/RBP2 SIM binds to SUMO antiparallelly in the context of the SUMO–RanGAP1–UBC9–Nup358/RanBP2 complex (40). Interestingly, a weak intramolecular interaction exists between the DAXX N-terminal intrinsically disordered region (aa 1–56) and the DAXX 4HB. This intramolecular interaction appears to interfere with the SIM1/SUMO interaction as well as the binding of p53, MDM2 and RASSF1C to 4HB (16). Phosphorylation of two serines (Ser-737 and Ser-739) in SIM2, mediated by casein kinase 2 (CK2), increases the binding affinity of SIM2 to SUMO1 by ∼30-fold. The phosphorylated SIM2 binds SUMO1 more tightly than SUMO2, thus conferring SUMO paralog selectivity (38). In general, phosphorylation in SIMs increases the numbers of negatively charged residues flanking the hydrophobic core and consequently enhances the binding affinity to SUMOs through electrostatic interactions with positively charged residues in SUMOs (38,41–43). These observations indicate that the SUMO-SIM interactions can be regulated by cell signaling-induced phosphorylation (42). Notably, the DAXX SIM1 contains two evolutionarily conserved phosphorylation sites (Thr-4 and Ser-7) amino terminal to the hydrophobic core. Thus, the interaction between the SIM1 of DAXX and SUMOs could also be regulated by phosphorylation, which nonetheless must be experimentally tested. Early studies show that DAXX’s localization to PML-NBs requires PML SUMOylation (11,12). The subsequent identification of SIM2 documents a critical role of the SIM2-SUMO interaction for recruiting DAXX to PML-NBs (14). In osteosarcoma Saos-2 cells, the colocalization of a DAXX double SIM mutant and a PML mutant lacking SUMOylation sites was severely impaired (6), further supporting the importance of PML SUMOylation and the SIM–SUMO interaction in tethering DAXX to PML-NBs. Notably, SIM2 has been shown to play a dominant role, while SIM1 appears dispensable, for DAXX’s recruitment to PML-NBs in several cell lines such as COS-1 and HeLa (14,44). The intramolecular SIM1–4HB interaction in DAXX (16) could mask SIM1, which potentially renders SIM1 unavailable for binding SUMOylated PML. Significantly, DAXX SIM2 mutations impair DAXX SUMOylation. Likewise, SUMO mutations that weaken the SIM–SUMO interaction also reduce DAXX SUMOylation (14). Thus, SIM2 inactivation interferes with DAXX-PML interaction by (i) disrupting the SIM2-SUMO interaction and (ii) reducing DAXX SUMOylation.

Interestingly, at least one functional SIM of DAXX is required for the interaction between DAXX and the SUMO E2 conjugating enzyme UBC9 (6). The UBC9 C93A mutant, a catalytic mutant that blocks the SUMO-UBC9 conjugation, still binds DAXX. However, mutation of His-20 in UBC9, a key residue for the high affinity noncovalent binding of SUMO to the backside of UBC9 (43,45), disrupts the DAXX-UBC9 interaction (6). Thus, DAXX appears to bind UBC9 via the high-affinity SUMO-binding site in the backside of UBC9 (6,43,45,46). These observations suggest that the SIMs in DAXX, and perhaps more generally in some SUMOylation substrates, mediate the substrate-SUMO-UBC9 ternary interaction, presumably to facilitate substrate SUMOylation and SUMO chain synthesis. The structural basis of the SIM-facilitated SUMOylation and chain formation was recently reviewed (46). Notably, SIM2 is critical for targeting DAXX to heterochromatin sites for depositing H3.3, which, interestingly, depends on UBC9, while SIM1 appears to strengthen DAXX's recruitment to chromatin sites (44). Shastrula et al. proposed that UBC9-mediated SUMOylation of unknown heterochromatin proteins mediates DAXX recruitment (44). However, the formation of DAXX SIM–SUMO-UBC9 ternary complex might be sufficient for DAXX recruitment to heterochromatins.

### The DAXX acidic domain

DAXX contains a long stretch of about 50 consecutive acidic residues immediately C-terminal to the HBD (Figure 1). This acidic domain is important for interacting with the C-terminal regulatory domain (CTD) of p53 enriched with lysine residues (47). The DAXX acidic domain plays an important role in DAXX-mediated gene repression, and the acetylation of the p53 CTD weakens its interaction with transcription repressors containing acidic domains such as DAXX (48). Notably, although diverse histone chaperones share little sequence identity, they commonly contain intrinsically disordered regions and acidic domains (49). The DAXX acidic domain appears to increase the binding affinity to the H3.3/H4 dimer (20,49). Nonetheless, precisely how the DAXX acidic domain contributes to its histone chaperone activity and other functions requires further investigation.

## DAXX OVEREXPRESSION IN TUMORIGENESIS, PROGRESSION AND TREATMENT RESISTANCE

**Figure 2.**
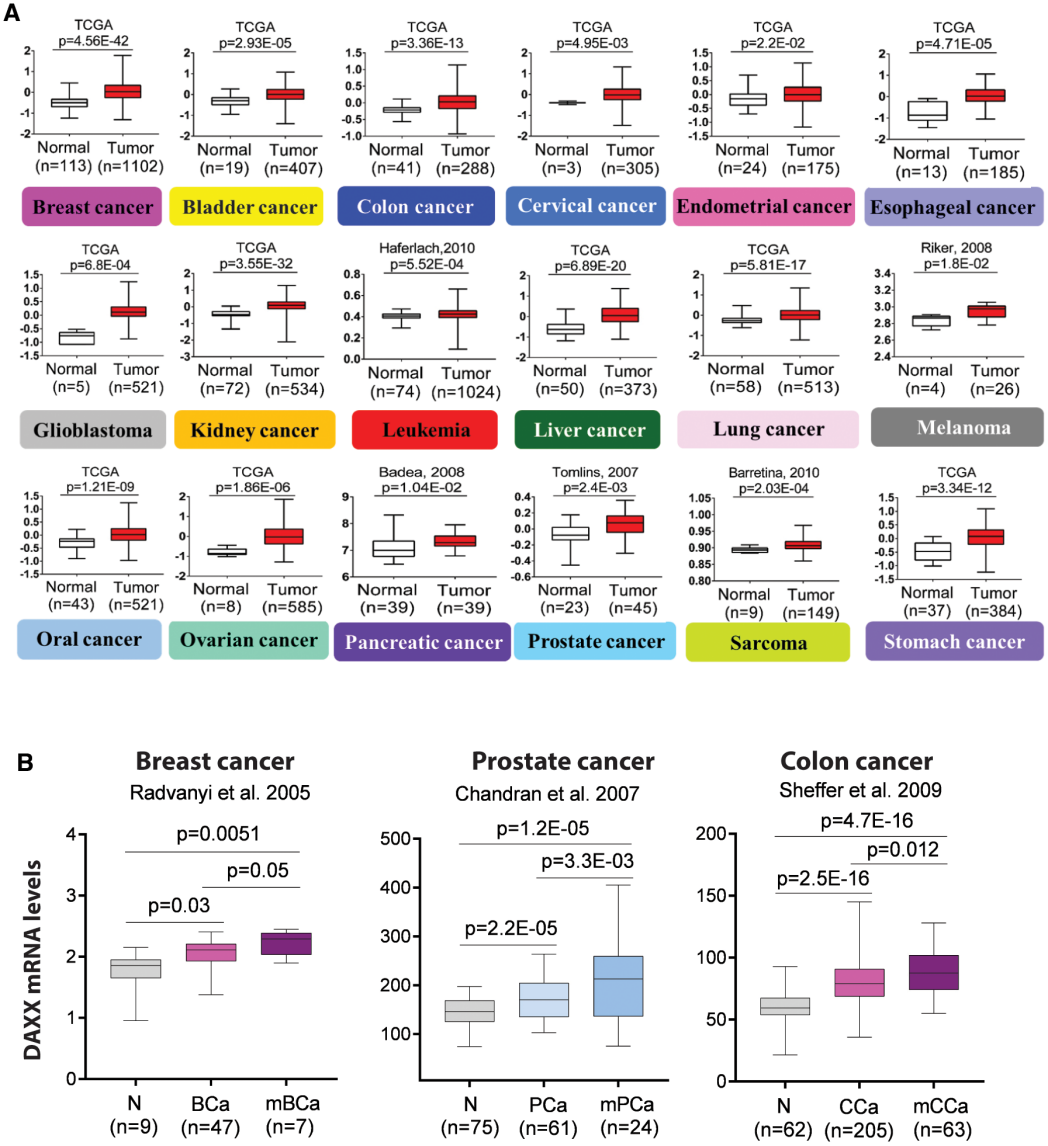
DAXX mRNA expression in cancer. (**A**) Boxplots depicting DAXX mRNA expression levels across multiple cancer types along with corresponding normal controls (data from the TCGA portal or other published datasets of clinical cancer samples as indicated). (**B**) DAXX mRNA levels are further elevated in metastases compared to primary tumors. BCa: breast cancer, mBCa: metastatic breast cancer, PCa: prostate cancer, mPCa: metastatic prostate cancer, CCa: colon cancer, mCCa: metastatic colon cancer.

The availability of gene expression data from diverse cancer types along with corresponding clinical parameters has greatly facilitated the identification of potential oncogenes. Our analyses of a large number of clinical samples from The Cancer Genome Atlas (TCGA) datasets along with other datasets of clinical cancer samples revealed markedly increased expression of DAXX in a variety of cancers compared to corresponding normal controls (Figure 2A). Significantly, DAXX levels are further increased in metastases compared to primary tumors in breast, prostate and colon cancers (Figure 2B). Additionally, immunohistochemistry analysis of tumor specimens has documented increased DAXX expression levels in several cancer types including prostate (50,51), ovarian (52), oral squamous cell carcinoma (53), and gastric cancer (54). In addition to the observed upregulation of DAXX in clinical cancer datasets, preclinical studies have provided compelling evidence supporting an oncogenic role for DAXX. DAXX’s activities in several biological processes, including cell death, cell survival, chromatin remodeling, gene regulation and DNA repair, may contribute to its oncogenic functions.

In ovarian cancer, DAXX was studied as a potential regulator of cell proliferation, metastasis, and drug resistance. Immunohistochemistry analysis indicates that DAXX levels are higher in different subtypes of ovarian cancer compared to normal control (52). Using cell lines and *in vivo* xenograft models, ovarian cancer cells with DAXX overexpression displayed enhanced tumorigenesis and metastasis *in vivo*, whereas DAXX depletion inhibited tumor development (52,55). Increased DAXX expression also resulted in heightened cell migration, invasion, resistance to chemotherapeutic agents and X-ray irradiation. In response to DNA-damaging agents, DAXX overexpression reduced, while its depletion increased, the number of γH2AX foci, suggesting that DAXX promotes DNA repair to protect cancer cells from DNA damage-induced cell death. In this context, DAXX and PML appear to work in concert to promote DNA repair. Recently, DAXX was shown to confer chemoresistance in ovarian cancer using both *in vitro* and *in vivo* models (56). Notably, the expression of adenovirus type 12 E1B-55 kDa protein, which interacts with DAXX (57,58), induces DAXX degradation, resulting in sensitization of ovarian cancer cells to cisplatin (56). Our analysis of a TCGA ovarian cancer dataset (59) shows that DAXX is highly upregulated in ovarian cancer samples compared with normal tissues (Figure 2A). Overall, these results suggest that DAXX upregulation might be a key step in ovarian tumorigenesis and disease progression and could be used as a diagnostic marker for ovarian cancers.

An interesting recent study reported that DAXX depletion in PTEN-null glioblastoma (GBM) cells impaired growth of orthotopically implanted tumors (60). DAXX interacts with PTEN and appears to antagonize PTEN-mediated repression of oncogenes. There is also an inverse correlation between DAXX and PTEN expression levels (60). It was suggested that altered H3.3 deposition in GBM cells deficient of PTEN but with high levels of DAXX results in oncogene expression (60). Interestingly, DAXX’s oncogenic property seems to be independent of ATRX in this setting (60). Consistent with an oncogenic function for DAXX in GBMs, our analyses of *s*everal GBM clinical datasets, including a TCGA GBM dataset (61), show that DAXX is significantly upregulated in GBMs (Figure 2A and data not shown).

In prostate cancer (PCa), DAXX promotes tumorigenesis and disease progression. DAXX is frequently elevated in PCa tissues, and the DAXX expression levels positively correlate with the Gleason scores and PCa metastasis (50,51). Interestingly, strong DAXX expression correlates with both TMPRSS2/ERG gene rearrangement and ERG expression (50). DAXX knockdown reduces xenograft tumor growth *in vivo*, apparently as a result of increased autophagy (62,63). DAXX interacts with the androgen receptor (AR) (64). Nonetheless, the functional ramifications of the DAXX-AR interaction in terms of AR-mediated transcription and prostatic oncogenesis remain unclear. Interestingly, DAXX interacts with the substrate recognition subunits (CDC20 and CDH1) of the E3 ubiquitin ligase anaphase promoting complex/cyclosome APC/C, which appears to inhibit the timely degradation of APC/C substrates, thereby potentially promoting chromosomal instability during PCa development (51). Nuclear exclusion of the tumor suppressor PTEN is linked to cancer progression. Monoubiquitination of PTEN is required for PTEN nuclear translocation (65). Interestingly, DAXX promotes USP7-mediated PTEN deubiquitination, resulting in its nuclear exclusion (66). Thus, increased expression of DAXX could interfere with PTEN nuclear localization, providing a mechanism for DAXX’s oncogenic function in PCa. As noted above, DAXX was shown to antagonize PTEN-mediated suppression of oncogene expression in GMB (60). Whether DAXX also opposes PTEN’s tumor suppression function in the nucleus in addition to blocking its nuclear import in PCa cells has not yet been examined. Our analysis of clinical PCa datasets (67,68) indicate that DAXX expression is elevated in PCa with further increase in metastases (Figure 2).

Notably, frequent mutations of the H3F3A gene encoding H3.3, and to a less extent, the HIST1H3B gene encoding the canonical histone H3.1 were identified in pediatric gliomas (69–71). The mutations most frequently occur at codons for K27 and G34 (69,71). Pediatric patients with diffuse intrinsic pontine glioma (DIPG) harboring the K27M mutation exhibits poor prognosis (72). Cells expressing H3.3K27M mutant display a global downregulation of H3K27me3 but upregulation of H3K27ac (73–75). However, although DAXX is a chaperone for H3.3, DAXX mutation is relatively rare compared to H3F3A and ATRX (69). Thus, whether and how DAXX is involved in tumorigenesis due to H3F3A mutations remains to be established. Interestingly, frequent mRNA upregulation of H3F3A and H3F3B (both encoding H3.3) is observed in diverse cancer types; gene amplification of both H3F3A and H3F3B is also common in some cancer types (our unpublished analyses). A recent study shows that H3.3 appears to metabolically stabilize DAXX (22). Therefore, increased H3.3 levels in cancer cells may augment DAXX’s oncogenic function through protein stabilization.

PanNETs are heterogeneous pancreatic neoplasms (76). Approximately 43% of PanNET cases have inactivating DAXX or ATRX mutations (21,77). DAXX/ATRX loss correlates with tumor stage, metastasis, and decreased survival (77–79). Several groups also reported that mutations of ATRX and DAXX in PanNETs impair the heterochromatic state of the telomeres, with reduced levels of histone variant H3.3 (23,24). Abnormal telomeres due to alternative lengthening of telomeres (ALT) independent of telomerase activity in PanNETs were observed frequently and can be attributed to the loss-of-function mutations of DAXX and/or ATRX (23,24,80). Interestingly, as noted above, DAXX mutations in PanNETs frequently occur to regions encoding the folded 4HB and HBD domains (Figure 1) (22), suggesting that the loss of DAXX’s H3.3 chaperone function may lead to abnormal chromatin structures, epigenetic dysregulation and chromosome instability. Recurrent mutations (frameshift and nonsense) of DAXX are observed in Hürthle cell carcinoma of the thyroid (81). SPOP was shown to promote kidney cancer, and SPOP-mediated DAXX degradation was proposed to contribute to kidney tumorigenesis (82). Nonetheless, DAXX mRNA levels are markedly elevated in kidney cancer (Figure 2). Therefore, further studies will be needed to determine how DAXX is involved in kidney tumorigenesis. Of note, active telomere maintenance mechanisms appear to correlate with worse prognosis for neuroblastomas, in which ATRX loss-of-function mutations may play a role in ALT. However, DAXX mutations were not detected in neuroblastomas. DAXX gene expression is also not significantly changed in ALT-positive neuroblastomas (83). Notably, SUMOylation of shelterin subunits (TRF1 and TRF2) and the recruitment of telomeres to PML-NBs are critical for telomere maintenance in ALT-positive cancer cells (84). Interestingly, in the osteosarcoma cell line G292, a chromosome translocation results in an in-frame fusion of DAXX with KIFC3. The DAXX exon 8 encoding SIM2 is lost. The fusion product is expressed in G292 cells but fails to localize in PML-NBs and to suppress ALT. The expression of wt DAXX in G292 cells inhibits ALT (85,86). These observations indicate that DAXX blocks ALT via PML-NBs.

Lin *et al.* reported that DAXX inhibits EMT, invasive growth phenotypes and metastasis through interfering with Slug-mediated repression of epithelial markers (87). These authors further showed that under hypoxia, hypoxia-inducible factor (HIF)-1α inhibits DAXX expression, resulting in increased invasion *in vitro* and metastasis to the lungs *in vivo* (87). Nonetheless, using a TCGA dataset based on clinical lung carcinoma samples, we found that DAXX expression is significantly higher in lung cancer compared with normal control tissues (88) (Figure 2). Our analyses of several other clinical data analyses also show upregulation of DAXX expression in different clinical types of lung cancer (data not shown). Further studies will be required to clarify roles of DAXX in lung cancer tumorigenesis vs. metastatic progression.

Overall, our data analyses described here indicate that DAXX is highly upregulated in diverse cancer types. Although somatic DAXX mutations are frequently observed in PanNETs (a rare cancer type), cancer-derived mutations in epidemiologically prevalent cancers are very rare. While published studies have documented an oncogenic role for DAXX, we still do not fully understand the molecular mechanisms underlying DAXX-mediated tumorigenesis and cancer progression. As discussed below, DAXX-mediated biological processes including transcription may contribute to DAXX’s oncogenic function. Given the prevalence of DAXX upregulation in cancer (Figure 2), a clear mechanistic understanding of DAXX’s oncogenic function may lead to widely applicable therapeutic strategies for treating many patients with cancer.

## DAXX-REGULATED PROCESSES

### DAXX in cell death and cell survival

#### Proapoptotic function of DAXX in the cytosol via the ASK1/JNK signaling pathway

**Figure 3.**
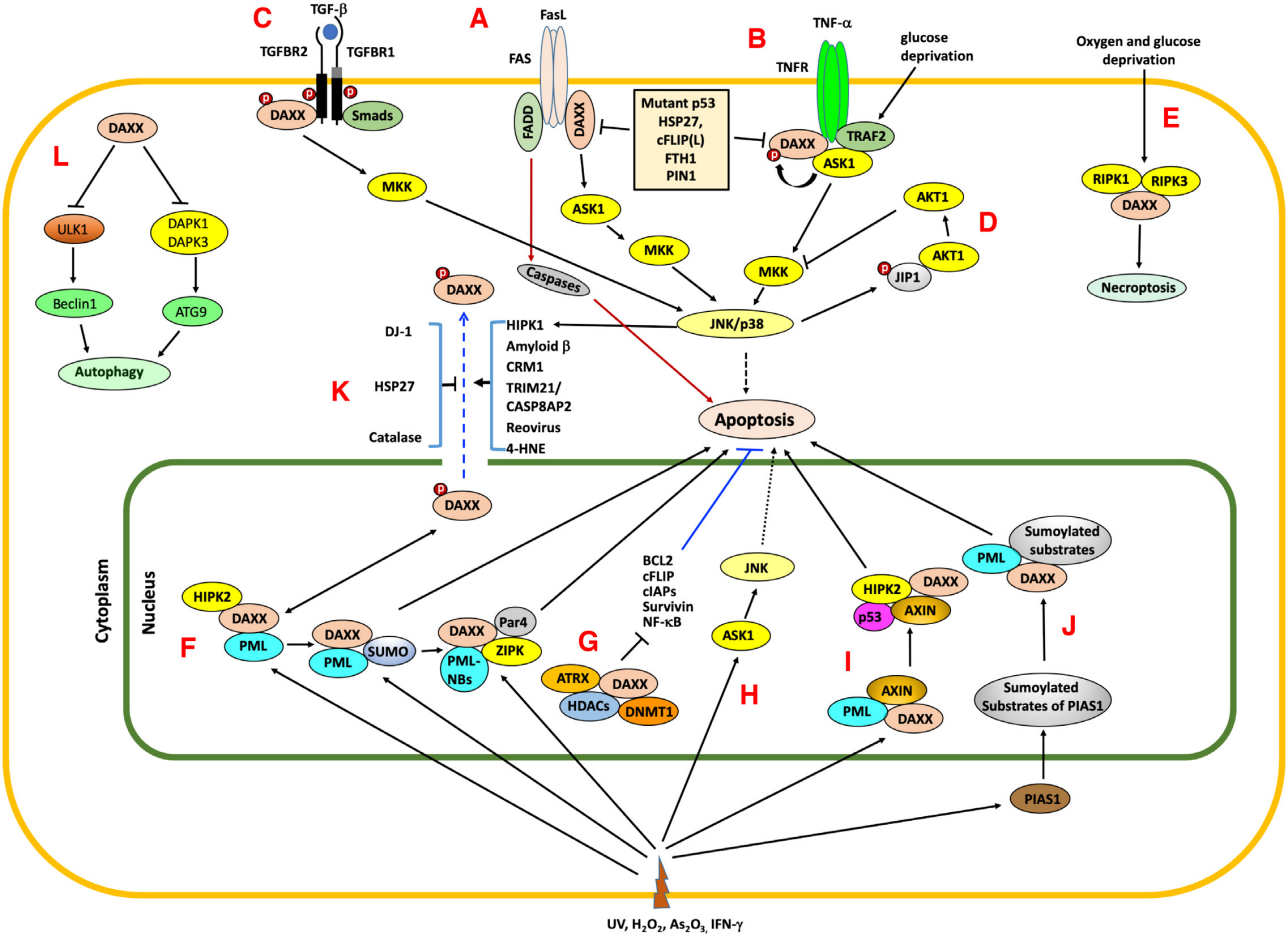
DAXX in cell death. Several ways in which apoptosis is induced by DAXX are shown. Induction of cell death by interacting with the death domain of Fas and other associated proteins, resulting in the activation of ASK1–JNK cell death signaling (**A** and **B**), and by TGFβ signaling, which mediates non-SMAD pathway activation (**C**). AKT1 blocks a cell death pathway mediated by DAXX through a negative-feedback loop (**D**). During necrosis in retinal ganglion cells, RIPK3 interacts with and phosphorylates DAXX (**E**). In the nucleus, DAXX-mediated cell death appears to be mediated by the PML-NBs, presumably by interacting with other proteins within PML-NBs (**F**). In the nucleus, DAXX can repress the expression of anti-apoptotic genes such as Survivin (BBC3) (**G**). DAXX activates the ASK1–JNK cascade in the nucleus upon UV exposure (**H**). DAXX can activate proapoptotic genes, for example, via p53 activation (**I**). In this case, the localization of DAXX and AXIN in PML-NBs may be important. DAXX seems to potentiate UV-induced apoptosis through modulating the SUMO E3 ligase PIAS1 activity (**J**). In general, cytoplasmic localization of DAXX has been described as a proapoptotic event, which can be induced by cellular proteins, viral infection and metabolites (e.g., 4-HNE), and blocked by cytoprotective factors (**K**). DAXX represses the expression of several components of autophagy machinery such as ULK1, DAPK1 and DAPK3, ultimately suppressing autophagic cell death (**L**). Cellular stresses such as the presence of interferons (IFNs), UV irradiation, and oxidative stress can trigger DAXX-mediated cell death events originated from cell surface receptors or regulation of gene expression in the nucleus.

DAXX is a pro-apoptotic protein associated with the death receptor FAS in the cytosol (4), and mediates apoptosis through the FAS-DAXX-ASK1-MAP2K axis to activate JNK and p38 mitogen-activated protein kinase (MAPK) cascades (5,89,90) (Figure 3A). A positive feedback loop between DAXX and ASK1 (apoptosis signal-regulating kinase 1) was observed, such that DAXX activates ASK1, which in turn phosphorylates DAXX (91) (Figure 3B). Cellular stress, such as glucose deprivation, activates the ASK1–MEK–MAPK signaling cascade via an association of DAXX and TRAF2 with ASK1 (92,93) (Figure 3B). JNK-driven HIPK1 activation is involved in the relocalization of DAXX from the nucleus to the cytoplasm, activating the DAXX–ASK1–JNK cell death pathway during glucose deprivation (92–94) (Figure 3B). Notably, AKT1 was reported to counteract the ASK1–JNK cell death pathway in a negative feedback loop (Figure 3D). By contrast, c-FLIP_L_, an inhibitor of caspase 8, binds to DAXX through interaction between the C-terminal domain of c-FLIP_L_ and the FAS-binding domain of DAXX to inhibit JNK activation by interfering with the interaction of DAXX and FAS (95) (Figure 3A).

A proapoptotic role for DAXX via the ASK1–JNK signaling pathway in the cytosol has been supported by multiple lines of evidence. In these studies, apoptotic stimulation triggers DAXX’s nuclear export to activate the ASK1–JNK axis (96); inhibiting this translocation by catalase (97), HSP27 (98–100), and DJ-1 (encoded by the PARK7 gene) (101,102) blocks apoptosis (Figure 3K). Interestingly, peptidyl-prolyl isomerase (PIN1) inhibits DAXX-mediated apoptosis by promoting DAXX degradation (103) (Figure 3A and B). Studies with transgenic mice expressing the DAXX C-terminal domain implicate a proapoptotic role *in vivo* for DAXX in T-cells (104) and cardiomyocytes (105). Notably, the expression of a full-length Daxx transgene in B cells impairs B cell proliferation but does not trigger apoptosis (106). Also, as discussed blow, specific Daxx knockout in T-cells compromises cell survival (107). Thus, how DAXX affects cell fates in the immune system requires further study.

#### DAXX in TGFβ-mediated apoptosis

DAXX interacts with the cytoplasmic domain of the type II TGFβ receptor (TGFBR2) and mediates TGFβ-induced apoptosis via JNK activation in the mouse hepatocyte cell line AML12 (108) (Figure 3C). TGFβ did not appear to increase DAXX phosphorylation but increased DAXX metabolic stability (108). Subsequently, homeodomain-interacting protein kinase 2 (HIPK2), involved in transcriptional regulation and apoptosis, was shown to activate JNK via DAXX and the mitogen-activated protein kinase kinases MKK4 (MAP2K4) and MKK7 (MAP2K7). HIPK2 colocalizes with DAXX in PML-NBs and induces DAXX’s release from PML-NBs. HIPK2 directly phosphorylates DAXX, which promotes TGFβ-mediated cell death (109). It is worth noting in this context that HIPK2 appears critical to glucose deprivation-mediated cell death via JNK activation (110). Given that DAXX cytoplasmic translocation and JNK activation are involved in inducing cell death during glucose deprivation (92,93), it will be interesting to assess whether HIPK2-mediated effects on DAXX is functionally linked to cell death upon metabolic stress. SMAD4 is a key mediator of the TGFβ signaling pathway. DAXX interacts with SUMOylated SMAD4 and represses SMAD-mediated transcription (111). SMAD4 activation induces growth arrest and apoptosis and its loss potentiates tumorigenesis (112). These observations suggest that DAXX could also block the tumor suppressive effect of TGFβ signaling by repressing SMAD4. Of note, RIPK3 phosphorylates DAXX, resulting in its cytoplasmic localization and necroptosis (113) (Figure 3E).

#### The proapoptotic function of DAXX in the nucleus

In the nucleus, DAXX can potentiate apoptosis through repressing the expression of antiapoptotic genes, activating proapoptotic genes and other less-well defined mechanisms (Figure 3F–J). DAXX has been shown to mediate cell death in response to oxidative stress (e.g. induced by arsenic trioxide), cytokines (TNFα and IFNγ) and osmotic stress, likely through repressing antiapoptotic genes (38,114) (Figure 3F and G). In the nucleus, DAXX was shown to activate the ASK1–JNK proapoptotic signaling in primary fibroblasts upon UV exposure (115) (Figure 3H). DAXX promotes p53-mediated apoptosis by repressing p21 expression in response to cisplatin treatment (116), and by activating the proapoptotic BCL2 family genes such as PUMA (encoded by BBC3) under UV irradiation (117) (Figure 3I). In the latter case, DAXX forms a complex with AXIN and HIPK2 to promote p53 phosphorylation at Ser-46, enhancing PUMA expression and apoptosis (117) (Figure 3I). Of note, a recent study showed that the mouse embryonic lethal phenotype due to homozygous Daxx knockout is unrelated to p53 activation (8). This suggests that the functional link between DAXX and p53 depends on cell types and biological contexts. PDCD4 interacts with DAXX and promotes DAXX degradation, resulting in reduced p53 Ser-46 phosphorylation in the absence of DNA damage. UV triggers PDCD4 downregulation, thereby restoring p53 Ser-46 phosphorylation (118). Of note, PDCD4 is an inhibitor of the translation initiation factor eIF4A, an RNA helicase that catalyzes the unwinding of secondary structure at the 5′ untranslated region (5′UTR) of mRNAs, thereby inhibiting cap-dependent protein translation (119). A potential broader functional link between DAXX and PDCD4 remains to be explored. DAXX interacts with substrates SUMOylated by PIAS1, and such interactions lead to UV irradiation-mediated apoptosis (120) (Figure 3J), although the mediators(s) of apoptosis in this context remains to be identified.

Induction of apoptosis by DAXX in the nucleus correlates with the localization of DAXX to the PML-NBs (114,121–123) (Figure 3F). SUMO modification is a prerequisite for PML to recruit DAXX to the PML-NBs (11,124). As discussed above, DAXX’s localization to PML-NBs may relieve its repression of proapoptotic genes (10,12,125), resulting in cell death. Notably, overexpression of SUMO-1 contributes to resistance to FAS-induced apoptosis through increased PML SUMOylation and increased recruitment of DAXX to PML-NBs. By contrast, the SUMO-specific protease SENP1 promotes the release of DAXX from PML-NBs (126). Thus, in this context, the localization of DAXX in PML-NBs plays a role in cell survival. These observations suggest that the effects of DAXX’s recruitment to PML-NBs on cell survival or death may depend on cell-type and biological contexts. Nonetheless, it remains to be determined precisely how the localization of DAXX in PML-NBs regulates apoptosis vs cell survival.

#### DAXX in cell survival

Genetic inactivation of *Daxx* results in embryonic lethality in mice (7,8). Increased levels of apoptosis were observed in *Daxx*−/− cell lines, indicating that *Daxx* is an essential gene for mouse embryonic development and plays a role directly or indirectly in preventing apoptosis (7,127). DAXX downregulation in human cancer cells sensitizes cells to apoptosis through the activation of JNK and caspases (128,129). In primary mouse ovarian epithelial cells, DAXX depletion accelerated senescence in a p53/p21-dependent manner and promoted DNA damage signaling (55). In p53-null osteosarcoma Saos-2 cells, DAXX downregulation correlates with caspase-3 activation and apoptosis (130,131). The lipid peroxidation (LPO) end-product 4-hydroxy-2-nonenal (4-HNE) induces a marked increase of DAXX in the cytoplasm, indicating that 4-HNE may facilitate the export of DAXX from the nucleus to the cytoplasm (Figure 3K). In this context, DAXX depletion seems to potentiate apoptosis (132). Interestingly, DAXX itself appears to be covalently modified by 4-HNE, although the functional consequence of this modification remains unknown.

Interestingly, DAXX is downregulated by HDAC inhibitors (133) and Berberine (134), whereas doxorubicin did not affect DAXX expression levels (134). In acute lymphoblastic leukemia (ALL) cells, Berberine appears to reduce DAXX mRNA levels, resulting in MDM2 degradation, and p53 activation (134,135). This indicates that DAXX-mediated p53 inhibition confers a cell survival advantage, in agreement with a previous study (47). In HeLa cells, DAXX sequesters RASSF1C in the PML-NBs. Upon DNA damage signaling, DAXX undergoes ubiquitin-dependent degradation, which releases RASSF1C from the nucleus to promote SAPK/JNK activation and cell death (136). In human TF-1 cells (a myeloid progenitor-derived cell line), overexpression of DAXX inhibits cell-extrinsic apoptosis, apparently through repressing AP-1-mediated transcription (137). Daxx down-regulation promotes, while its overexpression inhibits, apoptosis in cardiomyocyte-like cells exposed to H_2_O_2_ or hypoxia, indicating a cytoprotective role for DAXX (138). In addition to its role in regulating apoptotic cell death, DAXX represses the expression of several autophagy regulators to block autophagy (62,63) (Figure 3K). *In vivo*, Daxx knockout in T-cells results in increased apoptotic cell death upon T-cell receptor stimulation, while Fas-induced apoptosis appears unaffected, indicating that Daxx is important for T-cell survival (107).

### DAXX in transcriptional regulation

DAXX has a well-documented role in transcriptional regulation. In most cell types, DAXX predominantly localizes to the nucleus and functions there as a transcriptional coregulator through its interactions with a growing number of transcription factors and other nuclear proteins (Figure 1 and Table 1). DAXX-mediated transcriptional repression of diverse target genes has been reported (9,38,64,111,139–141). Likewise, studies have also shown that DAXX is involved in the activation of transcription (6,53,142–146). DAXX-regulated genes are probably important effectors in cell death, survival, and tumorigenesis.

**Table 1.** DAXX interacting proteins

DAXX-binding proteins	Method of study	Functions	References
FAS	Y2H, Co-IP	Apoptosis	(4)
CENP-C	Y2H, IF	Centromere maintenance and cell death	(197)
CENP-B	Co-IP, IF	H3.3 deposition at the centromeres	(198)
ATRX	Co-IP, IF, gel filtration chromatography, crystallography	Chromatin remodeling, transcription repression, H3.3 deposition	(22,27,28,32,199)
ASK1 (MAP3K5)	Co-IP	ASK1 activation and apoptosis	(5,176)
RIPK3	Co-IP	DAXX phosphorylation at Ser-668 and cell death	(113)
HIPK1	Co-IP, GST pulldown, IF	DAXX phosphorylation, DAXX nuclear export, apoptosis	(92,178)
HIPK2	Co-IP, GST pulldown, IF	DAXX phosphorylation, JNK activation, apoptosis	(109)
HIPK3	Co-IP	DAXX phosphorylation	(200)
PML	Co-IP, Y2H, IF	Apoptosis, transcriptional regulation	(11,12,121,122)
SUMOs	Y2H, IF, Co-IP, GST pull-down	DAXX localization to PML body, transcriptional regulation	(6,11,14,38,201)
UBC9 (UBE2I)	Y2H, Co-IP, GST pulldown	Not determined	(6,201)
TGFβRII	Y2H, GST pulldown, Co-IP	TGFβ-induced apoptosis	(108)
DAPK3 (ZIPK)	Co-IP	Apoptosis through PML-NBs	(114)
AXIN	Y2H, Co-IP	p53 activation and apoptosis	(117)
HSP27	Y2H, Co-IP, GST pulldown	Cell survival	(202)
TOLLIP	Y2H, IF, GST pulldown	Not determined	(203)
DJ-1 (PARK7)	Y2H, Co-IP, GST pulldown	Inhibits DAXX export to the cytoplasm, protects against DAXX/ASK1-induced cell death	(101)
PIN1	GST pull-down, Co-IP, IF	Cell survival, DAXX degradation	(103)
cFLIP_L_	Co-IP	Inhibits JNK activation	(95)
FTH1	Y2H, Co-IP, GST pull-down	Inhibits apoptosis	(204)
CRM1	Co-IP	DAXX nuclear export	(94)
TSG101	Co-IP	Transcription repression	(205)
PDCD4	Co-IP, GST pulldown	DAXX degradation	(118)
PIAS1	IF	Promotes ultraviolet (UV)-induced apoptosis	(120)
PTEN	Co-IP	Represses oncogene expression	(60)
p53, p73, p63	Y2H, Co-IP, GST pulldown, NMR	Cell death/survival	(116,117,25,47,206)
ETS1	Y2H, GST pulldown, IF	Transcription repression	(139)
Pax3	Y2H, Co-IP	Represses PAX3-mediated transcription	(9)
Pax7	Y2H	Not determined	(9)
Pax5	Y2H, Co-IP, GST pulldown	Transcription repression or activation	(142)
RELB (NF-κB)	Co-IP	Transcription repression	(207)
RELA (p65) (NF-κB)	Co-IP, GST pulldown	Inhibits p65 acetylation and transactivation	(141)
HSF1	Y2H, Co-IP	Activates HSF1-mediated transcription	(143)
SMAD4	Y2H, Co-IP	SUMOylation-dependent transcription repression	(111)
SNAI2 (Slug)	Co-IP	Suppresses SNAI2-mediated gene repression and inhibits metastasis	(87)
AIRE	Y2H, Co-IP, IF	Represses AIRE-mediated transcription	(208)
DNMT1	Y2H, Co-IP	Transcription repression, promoter methylation	(170,205)
DNMT3a	Co-IP	Not determined	(170)
DMAP1	Y2H, Co-IP, IF	Transcription repression	(205)
HDAC1, HDAC2, HDAC3	Co-IP, GST pulldown	Transcription repression	(12,13)
CBP	Co-IP	Represses or activates CBP-dependent transcription	(140,142)
AR	Y2H, Co-IP, GST pulldown	Inhibits AR-mediated transcription	(64)
GR	Y2H, GST pulldown	Transcription repression	(209,210)
MR	Y2H	Transcription repression	(210)
TCF7L2 (TCF4)	Y2H, Co-IP	Transcription repression or activation	(145,53,144)
STAT3	GST pulldown	Represses STAT3-mediated transcription	(211)
Histone H3.3/core histones	Co-IP, GST pulldown, crystallography	Histone chaperone	(13,19,20,26,212)
DEK	Co-IP	Not determined	(13)
BRG1	Co-IP	Not determined	(33)
MENIN	Co-IP	Gene repression and tumor suppression	(213)
MSP58 (MCRS1)	Y2H, GST pulldown, Co-IP, IF	DAXX nucleolar localization, reversal of DAXX-mediated gene repression	(214)
MDM2	Co-IP, NMR	Promotes p53 ubiquitination and degradation	(25,47,148)
RASSF1A	Co-IP	Disrupts DAXX-MDM2-USP7 complex and promotes MDM2 degradation	(66)
RASSF1C	Co-IP, NMR	RASSF1C nuclear sequestration	(25,136)
CDC20	Co-IP	Inhibits mitotic progression	(51)
CDH1	Co-IP	Inhibits mitotic progression	(51)
MAD2	Co-IP	Not determined	(51)
BUBR1	Co-IP	Not determined	(51)
CHIP (STUB1)	Co-IP	DAXX ubiquitination; inhibition of p53-mediated apoptosis	(184)
TRIM21 (Ro52)	Y2H, Co-IP, IF	DAXX cytoplasmic localization	(215)
GLUT4	Y2H, Co-IP	Not determined	(216)
Viral proteins that interact with DAXX			
E1B 55-kDa (HAdV)	Y2H, Co-IP, IF	Relocation of DAXX from PML-NBs	(57)
Protein VI (HAdV)	Co-IP, IF	Inhibits DAXX-mediated repression of viral gene expression	(217)
LANA (KSHV)	Co-IP, IF, GST pulldown	Inhibits DAXX-mediated transcriptional repression	(218)
BNRF1 (EBV)	Co-IP, gel filtration chromatography	Reverses DAXX/ATRX-mediated repression of viral gene expression; promotes latent viral gene expression	(169,219,220)
pp71 (HCMV)	Y2H, Co-IP, IF	Inhibits DAXX-mediated repression of viral gene expression	(221,222)
L2 (HPV)	Co-IP, IF	Blocks PML-NB-mediated repression of viral gene expression	(223,224)
Integrase (ASV, HIV)	Y2H, Co-IP, IF, GST pulldown	Represses viral gene expression	(159,162)
DENVC (Dengue virus)	Co-IP	Induces apoptosis	(166)
PUUV-N (Hantavirus)	Y2H, Co-IP, IF, GST pulldown	Not determined	(225)

Abbreviations: ASV: avian sarcoma virus; Co-IP: co-immunoprecipitation; EBV: Epstein Barr virus; GST: glutathione S transferase; HAdV: human adenovirus; HCMV: human cytomegalovirus; HPV: human papillomavirus; IF: immunofluorescence; KSHV: Kaposi's sarcoma-associated herpesvirus; LANA: latency-associated nuclear antigen; NMR: nuclear magnetic resonance; Y2H: yeast two-hybrid assay

### DAXX in DNA damage response (DDR)

DAXX is implicated in DDR in several ways. First, DAXX is involved in DNA-damage-induced p53 activation. RASSF1A, a DAXX-binding protein, destabilizes MDM2 by disrupting the MDM2–DAXX–USP7 (HAUSP) interactions, contributing to DNA damage-induced p53 activation (147). Ataxia telangiectasia mutated (ATM) regulates the DAXX-MDM2-p53-USP7 interactions (148). ATM phosphorylates DAXX at Ser-564 upon DNA damage (149,150). As noted above, DAXX may be involved in promoting DNA repair in ovarian cancer cells in response to genotoxic insults (52). These findings provide evidence to support a role for DAXX in DDR. Interestingly, the p53-regulated phosphatase WIP1 (PPM1D) dephosphorylates pSer-564 of DAXX (150). Notably, DNA damage-induced DAXX Ser-564 phosphorylation does not seem to affect p53 stability or the expression of p53 target genes in the human osteosarcoma cell line U2OS and primary human BJ fibroblasts (150). However, it is important to note that the U2OS cell line is deficient of ATRX. It will be interesting to assess whether ATRX plays a role in DAXX-mediated regulation of p53 in DDR.

Second, DAXX and ATRX protect DNA replication fork. Earlier studies show that inactivation of Atrx in mice results in p53 activation and neuronal cell death (151). Proliferating cells lacking Atrx exhibit delayed S-phase progression along with elevated DNA-damage stress, followed by cell death in rapidly expanding progenitors of several tissue types including the central nervous system (CNS) (151–154). Nonetheless, whether DAXX works together with ATRX in these settings remains to be investigated. Experiments using HeLa cells depleted of ATRX or DAXX indicate that ATRX/DAXX functions to protect stalled replication fork by limiting MRE11-mediated DNA degradation (154).

Third, DAXX deposits H3.3 at sites of new DNA synthesis in repairing double-strand breaks (DSBs) via homologous recombination. Recently, Juhasz et al. showed that cells depleted of ATRX, DAXX, or H3.3 exhibit identical defects in homologous recombination. DAXX together with ATRX acts to deposit H3.3 during repair DNA synthesis (155). Thus, the histone chaperone function of DAXX is directly linked to DNA repair. It will be interesting to investigate whether DNA damage-induced DAXX phosphorylation (e.g. at Ser-564) regulates its histone chaperone activity, thereby impacting DNA repair.

### DAXX in viral infection

Through interacting with viral proteins, DAXX represses viral gene expression, which is thought as an innate host antiviral response (57,156–162). Viral factors in infected cells counteract this antiviral mechanism by targeting DAXX for proteasomal degradation (58,161) or by disrupting the PML-NBs (57,163,164). DAXX can also confer antiviral immunity through triggering cell death of infected wells (164). For example, Daxx expression is markedly increased in the reovirus-infected mouse brain tissues through the type I interferon system, which triggers Daxx-Fas colocalization in plasma membranes, suggesting that Daxx mediates Fas-induced cell death (165) (Figure 3A). Surprisingly, endogenous Daxx also appears to repress caspase 3 expression to inhibit apoptosis in the reovirus-infected cells (165). These findings suggest that the cytoplasmic localization of Daxx is critical for Daxx to trigger apoptosis, while nuclear Daxx inhibits cell death through repressing proapoptotic genes to protect cells. There is also evidence that viral factors inhibit DAXX-mediated cytoprotection, which could facilitate the release of viral progenies (166,167). DAXX has also been shown to promote viral gene expression (168,169). The Epstein-Barr virus (EBV) tegument BNRF1 interacts with the DAXX–H3.3/H4 complex via directly contacting all three subunits of the DAXX–H3.3–H4 complex, which promotes viral latency and host cell immortalization (169).

## MECHANISMS UNDERLYING DAXX-MEDIATED PROCESSES

### Mechanisms underlying DAXX-mediated transcriptional regulation

A ChIP-seq study revealed DAXX’s chromatin-binding sites in promoters of actively transcribed genes, intronic and intergenic regions. The binding motifs of AP-1, nuclear receptors (NR), and FOXA1 are highly enriched in DAXX-associated chromatins (62). Consistent with previous studies (170), DNMT1 co-occupies a small subset of the DAXX-binding sites, which correlates with gene repression (62). The AP-1 motif is also enriched in sites co-bound by DAXX and DNMT1. Along with findings that DAXX promotes c-JUN-mediated transcription (6), these data indicate that c-JUN may play a major role in DAXX-mediated transcriptional regulation. As noted above, DAXX inhibits autophagy by repressing genes involved in this process, thereby promoting prostatic tumorigenesis (62,63).

**Figure 4.**
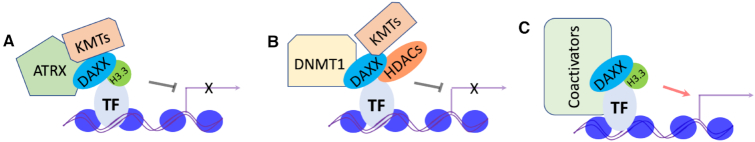
Mechanisms underlying DAXX-mediated transcriptional regulation. (**A**) DAXX, as an H3.3 chaperone along with ATRX, is recruited to chromatin by interacting with a TF. This complex deposits H3.3 to specific chromatin sites. Histone methyltransferases (KMTs) such as SETDB1 and SUV39H1 are associated with the DAXX repression complexes and mediate H3K9 trimethylation to repress transcription. (**B**) DAXX interacts with HDAC1, HDAC2 and HDAC3 as well as DNMT1 to repress transcription. KMTs such as SETDB1 are associated with the DAXX-HDAC1 repression complex. (**C**) DAXX can activate transcription through interacting with a TF, and possibly also with coactivator(s) such as CBP. H3.3 deposition may also be involved in DAXX-mediated gene activation. Multivalent interactions, partly mediated by the two SIMs of DAXX, are probably important for DAXX-mediated transcriptional repression and activation (not depicted).

Mechanistically, DAXX represses transcription through several distinct mechanisms. Daxx in concert with Atrx deposits H3.3 to certain endogenous retroviral elements. This leads to H3K9 methylation and transcription silencing (171) (Figure 4A). In hypomethylated genome, Daxx and Atrx were shown to occupy repetitive DNAs including telomeres to repress transcription by recruiting SUV39H1 to catalyze H3K9 trimethylation (172). As noted above, DAXX can tether DNMT1 to gene regulatory elements, resulting in increased DNA methylation and gene repression (62,170) (Figure 4B). Through interacting with HDACs (12,13), DAXX can mediate histone deacetylation at specific chromatin sites by binding to TFs (Figure 4B). Interestingly, DAXX, via the DAXX–SETDB1–KAP1–HDAC1 complex, represses endogenous retroviral elements, which does not involve ATRX and H3.3 deposition (22). Notably, DAXX interacts with the DNA-binding domain of several TFs including TCF4 (145), CREB (173), and Slug (SNAI2) (87), which interferes with DNA-binding of these TFs, thereby indirectly affecting gene expression. The interactions of DAXX with TCF4 or CREB result in reduced gene expression probably due to reduced chromatin binding of both TFs. However, DAXX forms a complex with Slug and HDAC1, which presumably is released from chromatin, thereby relieving gene repression by the Slug–HDAC1 complex (87).

DAXX can activate gene expression. As noted above, the AP-1 binding motif is highly enriched in DAXX-associated chromatin sites (62) and DAXX markedly activates c-JUN-mediated transcription in a SIM-dependent but PML-independent manner (6). DAXX depletion by an shRNA results in many downregulated genes along with upregulated ones (62). Mechanistically, H3.3 deposition was shown to activate certain genes (146). Interestingly, calcineurin-mediated dephosphorylation of the mouse Daxx at Ser-669 correlates with its chromatin association, H3.3 loading and transcriptional activation (146). Whether or not ATRX is involved in H3.3 loading at the gene regulatory regions was not examined in this study, although previous studies show that H3.3 deposition at those sites may be partly ATRX-independent (174). It will be important to identify factors (e.g., the histone chaperone DEK, as hypothesized previously (175)) that work with DAXX to deposit H3.3 at regulatory regions to activate gene expression (Figure 4C). The DAXX-CBP interaction is implicated in transcription activation (142), although the interactions of SUMO-1-modified CBP with DAXX and HDAC2 repress transcription (140). The functional outcome of the DAXX-CBP interaction may be context dependent. Collectively, DAXX-mediated transcriptional activation may involve its H3.3 chaperone function and coactivator recruitment (Figure 4C). Further studies will be required to define the regulatory dynamics of DAXX-mediated transcriptional activation and repression, which is likely regulated by specific cell signaling events.

### Molecular interactions impacting DAXX-mediated apoptosis

DAXX may serve as a scaffolding protein to form specific complexes to induce apoptosis. Upon Fas stimulation, DAXX binds to the N-terminal domain of ASK1, which disrupts the autoinhibitory intramolecular interaction of ASK1, resulting in the activation of the ASK1–JNK proapoptotic signaling cascade (5). Notably, K63-linked polyubiquitination of DAXX at K122 was shown to be important for TNFα-induced ASK1 activation (91) (Figure 3A and B), which possibly enables the assembly of a signaling complex akin to other cytoplasmic complexes associated with death receptors. In the nucleus, DAXX forms complexes with HIPK2, p53 and AXIN to facilitate p53 phosphorylation (117) (Figure 3I). Further studies are required to establish DAXX-containing complexes with defined composition and functions.

## REGULATION OF DAXX FUNCTIONS

DAXX undergoes extensive posttranslational modifications including phosphorylation, acetylation, ubiquitination and SUMOylation (www.phosphosite.org). DAXX protein abundance is regulated by ubiquitin-mediated proteasomal degradation. These events likely play critical roles in regulating DAXX’s biological functions.

### Phosphorylation

There are numerous sites of phosphorylation throughout DAXX, especially in the intrinsically disordered regions (Figure 1). ASK1 phosphorylates DAXX at Ser-176 and Ser-184, which stabilizes DAXX (176) and promotes K63-linked polyubiquitination of DAXX at K122 and cell death (91). By contrast, DAXX phosphorylation at Ser-178 is implicated in triggering DAXX polyubiquitination and degradation (103). Upon DNA damage, ATM rapidly phosphorylates DAXX at Ser-564 (149,150), which appears to promote p53 activation (149). Ser-712 was identified as a putative ATM/ATR phosphorylation site (177). RIPK3 was shown to phosphorylate DAXX at Ser-668 to promote DAXX nuclear export and cell death (113). CK2 phosphorylates DAXX at Ser-737 and Ser-739 within SIM2 to enhance its affinity to SUMOs (38). HIPK2 phosphorylates DAXX, which potentiates DAXX-mediated apoptosis (109). HIPK1 was shown to phosphorylate mouse Daxx at Ser-669 (178).

### SUMOylation and PML-NB localization

DAXX is SUMOylated at multiple sites, which is increased by oxidative stress (14,38). As noted above, multivalent interactions via numerous SUMO-SIM interfaces between DAXX and PML allow their high affinity interactions and the recruitment of DAXX, along with other components into PML-NBs, which may aggregate into phase-separated droplets (125,179,180). Interestingly, a DAXX mutant with 15 lysine to arginine mutations (15KR) that is largely devoid of detectable SUMOylation (14) can still localize to PML-NBs (14), indicating that the DAXX SUMOylation *per se* is not essential to its PML-NB localization. This is consistent with the observations that the two DAXX SIMs are the major determinant of DAXX’s PML-NB localization (6,14). Functionally, the localization of DAXX in PML-NBs has been suggested as a means to sequester DAXX to relieve repression of certain genes (10,12,125). As mentioned above, PML-NBs may also facilitate the deposition of H3.3 by DAXX to certain chromatin sites (31). Interestingly, PML blocks DAXX-mediated H3.3 incorporation into heterochromatin during S phase (44). Although precisely how the diverse functions of DAXX are regulated through PML-NBs remains to be established, PML-NBs may facilitate posttranslational modifications (phosphorylation, SUMOylation, acetylation and deacetylation) of DAXX and other clients (180). Indeed, CK2 and HIPK2 that phosphorylate DAXX localize to PML-NBs (38,109,180). Because CK2-mediated SIM2 phosphorylation increases its affinity to SUMOs (38), this can in turns promote DAXX SUMOylation. Through interacting with the PML N-terminal RING domain, UBC9 is concentrated in PML-NBs. It has been proposed that PML-NBs may facilitate SUMOylation of clients (180). However, whether or not DAXX is SUMOylated in PML-NBs remains to be established.

### Ubiquitin-mediated DAXX degradation

As discussed above, DAXX undergoes ubiquitin-mediated degradation in response to DDR and other stimuli (134,136). A number of E3 ubiquitin ligases have been identified to mediate DAXX proteasomal degradation including SPOP/CUL3 (18,82,181–183) and CHIP (184). Lys-630 and Lys-631 of DAXX are sites of CHIP-mediated ubiquitination, which appears to interfere with DAXX SUMOylation (184). SPOP-mediated DAXX degradation is best understood and potentially important for tumor suppression. Somatic SPOP missense mutations are frequently detected in several cancer types including endometrial and prostate cancer (185–187). Via the substrate-binding meprin and TRAF homology (MATH) domain, SPOP binds to the SPOP-binding (SB) motif with the consensus sequence of Φ-Π-S-S/T-S/T, where Φ is a nonpolar and Π is a polar residue (182). SPOP contains two dimerization domains, the BTB (broad-complex, tramtrack, and bric-a-brac) and the BACK (BTB and C-terminal kelch) domains, which mediate further oligomerization to form phase-separated liquid droplets (188). DAXX contains multiple SBs within its intrinsically disordered regions (Figure 1). The multiple weak DAXX-SPOP interactions in the C-terminal disordered region are critical to sequestering DAXX in the SPOP droplets to enhance DAXX ubiquitination (18). The SPOP-DAXX compartment is distinct from PML-NBs and nuclear speckles. Significantly, cancer-derived SPOP mutations impair SPOP-DAXX colocalization (18). Thus, cancer-associated SPOP mutations compromise its substrate recognition and the degradation of an increasing number of known and potential oncoproteins such as DAXX (18,181,182), BRD4 (189,190), MYC (191), the androgen receptor (AR) (192), PD-L1 (193), ERG (194) and Nanog (195,196).

### SUMMARY AND PERSPECTIVES

The literature reviewed here highlights critical functions of the ubiquitously expressed DAXX in cell survival, apoptosis and oncogenesis. As a chromatin-associated transcriptional regulator, DAXX modulates gene expression through binding to TFs, epigenetic modifiers, and chromatin remodelers. The genes regulated by DAXX are likely important mediators or effectors of DAXX’s biological functions. The regulation of other functions such as cell death by DAXX may also be through direct physical interactions between DAXX and various proteins. An understanding of signaling pathways that regulate DAXX-mediated biological processes will be informative to illuminate the context-dependent roles of DAXX in transcription, cell survival and apoptosis. Importantly, the compelling evidence that DAXX has an oncogenic function as reviewed here suggests that targeting of DAXX-mediated mechanisms could have therapeutic potential for treating different cancer types. A precise mechanistic understanding of how DAXX modulates gene expression, epigenetic modification, chromatin remodeling and DNA repair can provide innovative ideas for designing anticancer therapeutic interventions. Future work based on conventional methodologies in molecular cell biology along with integrated systemic OMIC approaches will undoubtedly yield more insights into the complex functions of DAXX in biology and pathobiology.
